# Using Continuous Glucose Monitoring and Data Sharing to Encourage Collaboration Among Older Adults With Type 1 Diabetes and Their Care Partners: Qualitative Descriptive Study

**DOI:** 10.2196/46627

**Published:** 2023-07-26

**Authors:** Alycia A Bristol, Michelle Litchman, Cynthia Berg, Ernest Grigorian, Denise Small, Ashley Glazener, Christopher Jones, Nancy A Allen

**Affiliations:** 1 College of Nursing University of Utah Salt Lake City, UT United States; 2 College of Social and Behavioral Science University of Utah Salt Lake City, UT United States; 3 College of Pharmacy Roseman University South Jordan, UT United States; 4 Cottonwood Medical Clinic Endocrine and Diabetes Intermountain Healthcare Murray, UT United States

**Keywords:** type 1 diabetes, continuous glucose monitoring, care partner, communication, data sharing, caregiver, caregiving, diabetes, diabetic, type 1, glucose, dyad, communication, older adult, elder, telehealth, collaboration, insulin, endocrinology, endocrine, self-efficacy, health education, insulin pump, tele, telehealth, hypoglycemia, hyperglycemia

## Abstract

**Background:**

Persons with diabetes use continuous glucose monitoring (CGM) to self-manage their diabetes. Care partners (CPs) frequently become involved in supporting persons with diabetes in the management of their diabetes. However, persons with diabetes and CP dyads may require more communication and problem-solving skills regarding how to share and respond to CGM data.

**Objective:**

The purpose of this study was to describe the experiences of persons with diabetes and CPs who participated in the Share “plus” intervention, which addresses dyadic communication strategies, problem-solving, and action planning to promote sharing of CGM data among the dyad.

**Methods:**

Ten dyads participated in the Share “plus” telehealth intervention. Participants were interviewed during and after the Share “plus” intervention. Thematic analysis was used to analyze interview data.

**Results:**

During postsession interviews, dyads described feeling a sense of shared responsibility yet viewed the persons with diabetes as ultimately responsible for the disease. Additionally, dyads shared that communication patterns improved and were able to recognize the negative aspects of previously established communication patterns. Dyads reported communication focused on hypoglycemia episodes while also differing in the frequency they reviewed CGM data and set alerts. Overall, dyads expressed positive reactions to the Share “plus” intervention.

**Conclusions:**

Share “plus” was helpful in promoting positive CGM-related communication among dyads and encouraged more CP support. CPs play an important role in supporting older adults with type 1 diabetes. Communication strategies help support dyad involvement in CGM data sharing and self-management among persons with diabetes.

## Introduction

### Background

The prevalence of type 1 diabetes (T1D) is estimated to affect up to 22 million individuals worldwide, with 1.6 million being aged 60 years or older [1]. The life expectancy of people with T1D has increased up to an additional 15 years, resulting in a higher incidence of older adults living with the disease [2,3]. Older adults living with T1D often experience age-related changes including increasing hypoglycemia accompanied by hypoglycemia unawareness [4]. Severe hypoglycemia in older adults can lead to loss of consciousness, seizures, falls, and other complications such as myocardial infarction [5-7]. However, technology such as continuous glucose monitoring (CGM) has recently been shown to be effective in reducing hypoglycemia and hyperglycemia in older adults with T1D [8,9]. Thus, the American Diabetes Association supports CGM in older adults with diabetes [10], which has become increasingly available to older adults after Medicare began covering this technology in 2017 [11].

Care partners (CP; eg, spouse, adult child, and friend) often want to be involved with the person with diabetes. Newer CGM apps that allow for glucose data sharing have the potential to facilitate the involvement of CPs in supporting persons with diabetes. Data sharing apps allow CGM readings and predictive hypo- and hyperglycemia alerts to be displayed on a CP’s smartphone or smartwatch via allowance from the primary user, free with a compatible CGM device. In the Diabetes Attitudes and Wishes Study, persons with diabetes reported a desire for family members to be more involved in their diabetes [12]. However, in the second Diabetes Attitudes and Wishes Study for family members living with a person with diabetes, family members reported they frequently lacked an understanding of how best to be involved while feeling burdened and distressed about diabetes and were worried about hypoglycemia [13].

Although CGM with data sharing holds promise for involving CPs in diabetes management, there are barriers to data sharing. These include the persons with diabetes not wanting to include others in their care, communication challenges between the person with diabetes and their CP and difficulties setting up the data sharing mobile apps. The challenges in communication often reflect persons with diabetes and the CPs’ different expectations regarding family involvement [14]. Persons with diabetes frequently regard diabetes as “their own illness,” whereas spouses view the illness as more shared [15,16]. Yet, when a person with diabetes and their spouse share the same appraisal that diabetes is “shared,” collaboration and support are more frequent [16,17]. When the spouse sees the illness as shared, there is an increase in self-care in persons with diabetes, likely by increasing their perceptions of greater emotional support and decreasing critical communication [17,18]. However, older adults are more likely to perceive diabetes as a shared condition than middle-aged adults [17]. Recent evidence from a couples-based intervention for those with type 2 diabetes [19] found that improving collaboration and communication supported quality of life benefits, as the intervention resulted in lower persons with diabetes and partner distress and higher relationship satisfaction. For those with moderately elevated glycated hemoglobin, the couples’ intervention led to improved glycemic levels.

In response to these barriers, we developed a multifaceted diabetes care and education intervention for older adults and their CPs. The SHARE “plus” intervention was delivered by telehealth and consists of a dyadic appraisal of diabetes, communication strategies, problem-solving strategies, and action planning. An assessment of the overall feasibility of the SHARE “plus” intervention is described elsewhere [20]. However, the rich interaction between dyads and the Diabetes Care and Education Specialist (DCES) represented a key component of the Share “plus” intervention. The purpose of this manuscript is to present findings from the dyadic conversations during the Share “plus” intervention and post intervention in an attempt to highlight the importance of supporting dyad communication during interventions targeting diabetes management.

### Dyadic Coping Model

**Figure 1:**
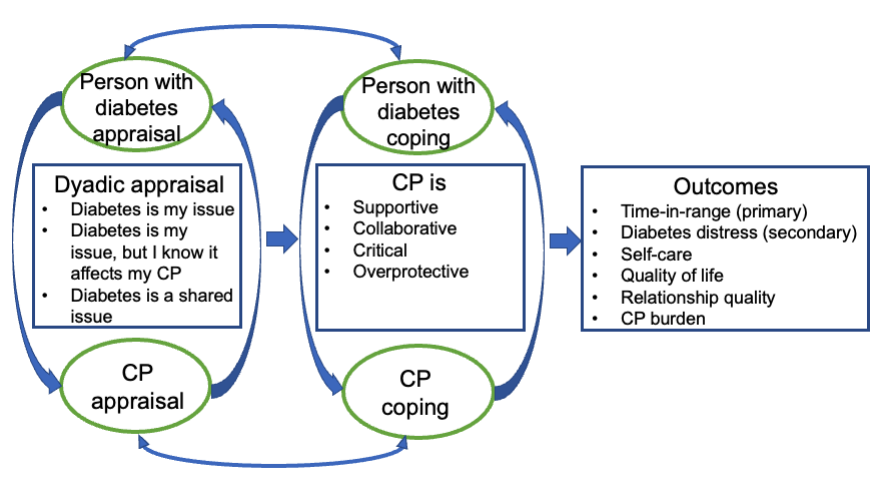
Dyadic coping process affected by Share “plus” intervention. CP: care partner.

The Dyadic Coping Model (DCM; Figure 1) posits that for persons with a chronic illness, dyadic coping can hold benefits for health outcomes, relationships, and the individual. Dyadic coping occurs when one individual perceives a stressor (in this case, diabetes) as “our” problem versus “my” or “your” problem and activates a process of collaborative coping to address stressors associated with diabetes [21]. Additionally, positive relationship quality and satisfaction between the persons with diabetes and CP can enhance self-management behaviors [22], and visibility of support [23]. However, collaborative involvement of the CP may be detrimental when the person with diabetes views diabetes as only their illness to deal with and does not consider its effects on the CP. In this study, the DCM was used in the development of dyadic education sessions focused on promoting the value of a CP’s collaborative involvement in the glucose monitoring of a person with diabetes via CGM and addressing the importance of supportive and unsupportive behaviors. Moreover, the DCM-guided organization of codes into categories during analysis.

## Methods

### Overview

This paper reports qualitative aspects from dyadic educational sessions as part of a larger intervention, the Share “plus” [20]. The Share “plus” intervention provides training to dyads in CGM communication and problem-solving and results in an action plan. Share “plus” includes evidence-based communication strategies, such as motivational interviewing questions, problem-solving, self-efficacy enhancement strategies, and action planning [10,24-26]. Full description of the Share “plus” intervention and results are reported elsewhere [27]. In this paper, we highlight participants’ experiences with the dyad intervention sessions with a DCES and postintervention feedback shared during follow-up interviews.

### Participants

Participants were recruited from an academic endocrinology specialty clinic and through social media flyers. Eligibility criteria included persons with diabetes who (1) aged ≥60 years, (2) diagnosed with T1D, (3) naïve to personal CGM use with the Dexcom Follow app, (4) glycated hemoglobin 6%-12% within the last 6 months, (5) able to read and write English, (6) own a smartphone compatible with the Dexcom G6 CGM, and (7) have a CP willing to participate. Persons with diabetes with or without an insulin pump were included. The Montreal Cognitive Assessment (MoCA) was used to screen participants [28]. The MoCA was performed as the incidence of dementia in persons with diabetes is more than 2 times that of people without diabetes [29]. Thus, we wanted to avoid the inclusion of individuals with moderate to severe dementia, as these individuals might lack the ability to participate in sessions targeting communication patterns. Persons with diabetes were excluded if they had (1) a MoCA score of <18, (2) a life expectancy estimated at <1 year, (3) unstable recent cardiovascular disease, significant malignancy, or other conditions resulting in physical decline, (4) a history of visual impairment that would hinder performing study procedures. Inclusion criteria for CPs were anyone identified by the persons with diabetes and (1) willing to use the Follow App, (2) willing to attend Share “plus” intervention education sessions, (3) were aged ≥18 years of age, (4) did not self-report cognitive impairment, and (5) owned a smartphone compatible with Dexcom Follow app. This study was conducted by telehealth with dyads in their own homes.

### Data Collection: DCES-Led Dyad Sessions

The DCES used evidence-based communication strategies, such as motivational interviewing questions, problem-solving, self-efficacy enhancement, and action planning. Motivational interviewing questions were used to help the dyads identify and strengthen their personal motivation for data sharing in a supportive conversation. For example, persons with diabetes were asked, “On a scale of 1-10 how would you rate your confidence in your ability to share your glucose numbers with your partner” and then “tell me a little bit about why you did not choose a higher score?” CPs were then asked the same questions. Several self-efficacy enhancing strategies were also used such as role modeling to describe the experiences of other persons with diabetes and CP’s that were similar. For example, dyads were asked how comfortable they felt about data sharing. Examples were provided about how other persons with diabetes have described the benefits of sharing their diabetes, such as an increased sense of teamwork, support, quality of life, and decreased diabetes-related burden. The barriers to sharing glucose levels were also identified (eg, glucose levels are private, persons with diabetes do not want to be judged). Verbal persuasion was used to provide education about effective and noneffective communication strategies. Past performance (eg, Mastery Experience) was also used to develop collaboration and knowledge. For example, if a person with diabetes reported that they viewed diabetes as their own illness, the dyad was asked to talk about something that they think of as shared such as planning a trip. Problem-solving action plan strategies included actions to take for hyper- and hypoglycemia and other CGM-related settings. Dyads were also asked to review problems that came up from the previous diabetes care and education session and generate solutions and options that might work moving forward. Last, several steps were taken to develop an action plan around agreed-upon communication strategies and CGM with data sharing settings and actions to take for hypo- and hyperglycemia (see Table 1).

**Table 1:** Overview of Share “plus” session topics and objectives.

Session and topic		Approach	Objective
**One**			
	Shared appraisal	Persons with diabetes and CP^a^ were asked to align themselves with 1 of 3 statements regarding their feelings about diabetes and its effects on their CP Persons with diabetes and CP rated their confidence on a scale of 1-10 regarding the sharing of blood glucose	To determine comfort level with data sharing for the persons with diabetes and their CP
	Communication	Persons with diabetes were asked how they would feel about sharing their low and high glucose levels Discussed helpful and unhelpful language	To explore supportive and unsupportive conversation strategies
	Problem-solving	Discussed problem-solving steps: identify the problem, find solutions, and take action (when needed) Identified concerns and willingness to problem solve the cause of low and high glucose levels.	To explore problem-solving of out-of-range glucose levels
	Action planning	Identified alarm settings for persons with diabetes and CP Agreed on how CP would contact the persons with diabetes for out-of-range blood glucose (call, text, etc) Confirmed supportive language for out-of-range glucose levels	To set clear expectations around data sharing
**Two**			
	Communication	Reviewed problems with data sharing Assessed if communication strategies were used Interventionist chose 1 or 2 communication strategies to discuss after listening to problems with data sharing	To further develop communication strategies
	Problem-solving	Reviewed helpful and unhelpful interactions Discussed frustrations with CGM^b^ setting Discussed CGM clarity data and impact of food on glucose levels	To explore glucose patterns and develop glucose management skills regarding the sharing of blood glucose levels and food choices
	Action planning	Agree on communication preferences around glucose levels and food choices and timing Confirmed problem-solving strategies around glucose levels Encourage routine discussions of glucose trends	To set communication preferences and to identify goals for dyadic problem solving of glucose levels
**Three**			
	Communication	Reviewed problems with data sharing and communication between the dyad Assessed if communication strategies were used	To develop dyadic communication strategies
	Problem-solving	Discussed CGM clarity data and effects of lifestyle (exercise, stress, illness) on glucose levels	To explore new problems about healthy eating and to develop glucose pattern management skills around exercise and stress
	Action planning	Agree on communication preferences around glucose levels and lifestyle behaviors Confirmed problem-solving strategies around glucose levels Encourage routine discussions of glucose trends with dyad using positive communication and effective diabetes management strategies	To set communication preferences around stress and exercise; to identify goals for dyadic problem solving of glucose levels

^a^CP: care partner.

^b^CGM: continuous glucose monitoring.

### Postsession Individual Interviews

Immediately after completing the 12-week Share “plus” sessions, dyads were invited to participate in individual interviews. Interview questions addressed dyad experiences and feedback regarding the Share “plus” sessions. Dyads were interviewed separately on Zoom (Zoom Technologies), by a trained research assistant and focused on using CGM, the Follow App (Dexcom), and the Share “plus” sessions.

### Rigor

Trustworthiness criteria from Lincoln and Guba [27] guided the rigor of this study [30]. A semistructured interview guide was used during the DCES dyad sessions and the postintervention interviews. DCES dyad sessions and interviews were recorded, transcribed verbatim, and verified for accuracy. AAB and NAA (lead authors) led the analysis as they have extensive background and experience in qualitative research. Team meetings were held where AAB, AG, DS, and NAA discussed and shared thoughts, reactions, and perceptions that emerged during data collection and analysis. Team members engaged in reflexivity throughout the analysis process, discussing previous experiences and personal perceptions that emerged during coding and theme development. A written record was maintained and comprised codes, definitions, decisions, memos, field notes, and team communication during the data collection and analysis processes. Team meetings were scheduled with the larger research team for feedback and input into the developed codes and themes. All participants of the Share “plus” intervention participated in the DCES dyad sessions and were invited to participate in the postintervention interviews. All but one dyad agreed to participate in the postintervention interviews.

### Ethics Approval

This study was approved by the University of Utah’s institutional review board (00114642). Informed consent was obtained and participants were informed of their right to opt out at any point of the study. Gift cards were given at the beginning of the study and at day 10 and at the end of the study for US $30 at each time point totaling US $90 for the person with diabetes and US $90 for the care partner.

### Analysis

Thematic analysis was used to develop major themes representative of the participants’ experiences during the DCES dyad sessions and postintervention interviews [31,32]. Coding followed 2 phases [33]. Phase 1 used inductive, open coding of the first 3 diabetic dyad sessions and the postintervention interviews and focused on describing behaviors outlined by participants. From this phase, a codebook was developed (see Table 2). During phase 2 of the analysis, team members AAB, DS, and AG (used the codebook to code the preceding sessions and Interview data. New codes continued to emerge and were added to the codebook. Codes were then organized into themes representing participants’ views of the key aspects of the Share “plus” intervention and influenced by concepts of coping and appraisal from the DCM. Team members met weekly to review coding and theme development. Disagreements were discussed until consensus and themes were reached.

**Table 2:** Selected sample of codebook.

Codes	Illustrative quote	Corresponding theme
Alarm sharing	*Ok, alerts go off, he knows, like when I’m high or when I’m low* [persons with diabetes]	Sharing and monitoring glucose data
View of role	*Because we’re married and we’re together and I’m concerned over whatever happens with him* [CP^a^]	Shared responsibility
Illness appraisal	*It is my issue, but I know it affects others. Because it actually impacts sometimes what I can do, you know?...it’s an inconvenience to me at times and becomes other people’s inconvenience as well* [persons with diabetes]	Independent appraisal of roles within dyads

^a^CP: care partner.

## Results

### Overview

Ten dyads met the recruitment criteria, and 100% of them completed the 3 Share “plus” sessions. One dyad did not complete the postintervention interview because of time constraints but did complete the 3 sessions. Demographics of the participants with diabetes and their CPs are listed in Table 3. The participants with diabetes, on average, were 66 (SD 4.78) years of age, and CPs were slightly younger (mean 62.8, SD 11.82 years). The sample was 100% White, and the majority had college degrees. Only one dyad had a parent-child relationship. MoCA scores were evaluated for all participants with diabetes prior to enrolling in the study. All participants had a MoCA score ≥ 26 except 2 individuals; 1 person with diabetes scored 25 and the other scored 22.

**Table 3:** Demographics for participants with diabetes and CPs (N=20).

Characteristics			Persons with diabetes (n=10; %)		CP^a^ (n=10; %)
Age (years), mean (SD)			66.8 (4.78)		62.8 (11.82)
**Sex, n (%)**					
	Female	5 (50)		6 (60)	
	Male	4 (40)		4 (40)	
	Prefer not to answer	1 (10)		0 (0)	
White race, n (%)			10 (100)		10 (100)
**Highest education, n (%)**					
	Associate degree or some college	0 (0)		1 (10)	
	Bachelor degree	3 (30)		2 (20)	
	Graduate degree	5 (50)		5 (50)	
	High school Graduate or general educational development	1 (10)		0 (0)	
	Vocational or technical school	1 (10)		2 (20)	
**Employment status, n (%)**					
	Disabled	0 (0)		1 (10)	
	Full-time	3 (30)		6 (60)	
	Part-time	0 (0)		2 (20)	
	Retired	7 (70)		1 (10)	
**Annual household income (US $)**					
	≤24,999	0 (0)		6 (60)	
	50,000 to 74,999	1 (10)		1 (10)	
	75,000 to 99,999	2 (20)		2 (20)	
	100,000 to 149,999	3 (30)		1 (10)	
	≥150,000	4 (40)		1 (10)	
	Declined to answer	1 (10)		4 (40)	
Type 1 diabetes			10 (100)		—^b^
Diabetes duration (years), mean (SD)			24.9 (21.66)		N/A^c^
**Relationship to persons with diabetes**					
	Child	N/A		1 (10)	
	Spouse	N/A		9 (90)	

^a^CP: care partner.

^b^Not available.

^c^N/A: not applicable.

### Results From DCES Dyad Session

#### Overview

Three themes developed representing experiences during the DCES dyad sessions include (1) independent appraisal of roles within dyads; (2) communication patterns; and (3) sharing and monitoring glucose data. During sessions with the DCES, the discussion focused on understanding the dyad’s baseline view of the role of the CP in supporting persons with diabetes and communication patterns. Working together with the dyad, the DCES was able to develop and tailor management strategies including how alarms were set and how the persons with diabetes and CP engaged with the CGM and Follow app.

#### Independent Appraisal of Roles Within Dyads

During the initial session with the DCES dyads were asked to consider how they viewed responsibility for diabetes management. Most persons with diabetes demonstrated a core sense of independence. For example, 1 person with diabetes stated, “It is my issue, but I know it affects others” (person with diabetes #4, aged 65 years), which the CP echoed by stating, “I think it's like it's her issue in the sense that only she can actively manage her diabetes. But there are lots of people who care about her well-being.” (CP #4, aged 67 years) While persons with diabetes expressed desire for self-control of management, CPs responses highlighted a desire to be in a supportive role.

The DCES also encouraged dyads to consider engaging in teamwork. Yet, when asked about teamwork, persons with diabetes echoed similar thoughts regarding independence. One person with diabetes stated, “Well, I guess because I’m the one with diabetes and she’s not. She just has to put up with me having diabetes and how it impacts our lives” (person with diabetes #3, aged 67 years). While the CP shared:

Regardless whether we’re together or apart, I still know basically how he’s doing and it affects me. I'm conscientious of what he should or shouldn’t be eating and watching what he does or how he's acting, especially if he is high or low, then it affects me in the middle of the night, the alarm goes off. And so if he's not sleeping, I'm not sleeping, and vice versa. So, I think it's very much a shared responsibility to make sure he's where he needs to be.

CP #3, aged 60 years

Overall, while the DCES introduced concepts of shared responsibility and teamwork, responses from persons with diabetes continued to center on the idea that disease management rested mainly with persons with diabetes, while CPs were more likely to be viewed as supportive partners only.

#### Communication Patterns

During sessions with the DCES, communication strategies were presented and reviewed. Initially, most dyads reported feeling they had previously established good communication patterns prior to participation in the Share “plus” intervention. One person with diabetes shared a commonly echoed sentiment, “We communicate pretty well. It's not going to change” (person with diabetes #2, aged 65 years). Dyads entered the Share “plus” intervention with strong feelings of having established good communication patterns. However, throughout the sessions, half of the dyads reported experiences with the use of unsupportive communication during hypoglycemic or hyperglycemic events. One person with diabetes shared, “I know I snap at him (CP) enough that he probably knows when you know when that happens” (persons with diabetes #5, aged 69 years).

Moreover, communication occurred primarily around hypoglycemic events. Dyads shared how hypoglycemic events were seen as more important, requiring communication. One person with diabetes shared:

The lows are a life, a life-threatening circumstance. The highs are not. But I would think so. Yeah, she's pretty much just minding your own business and keeping it to herself unless she feels that there’s a need to say something and the need would be a low alert, probably.

Person with diabetes #7, aged 73 years

Persons with diabetes recognized and appreciated CPs ability to communicate and support them during hypoglycemic events, while hyperglycemic events were seen as mainly the responsibility of the persons with diabetes. Dyads voiced concerns that there were limited options they could take when the persons with diabetes experienced hyperglycemia, which decreased communication around higher glucose trends. One CP shared:

I don't think we're educated enough to …actually come up with a solution to the problem. There’s just something that happens that we have no …way of dealing with it other than just waiting it out.

CP #9, aged 70 years

Communication patterns between dyads centered on reacting to hypoglycemia. While dyads often shared experiences with unsupportive communication, they overwhelmingly felt they had already established good communication patterns.

#### Sharing and Monitoring Glucose Data

Overall, persons with diabetes and CPs shared different approaches toward monitoring glucose trends at the start of the intervention. Persons with diabetes shared that they checked their levels multiple times throughout the day. One person with diabetes stated checking CGM data, “every hour or so” (person with diabetes #1, aged 67 years). In contrast, CPs reported a lower baseline engagement in monitoring glucose trends. One shared:

I don't really look at it that often...(persons with diabetes) is…quite capable on her own of, you know, monitoring your blood sugars and looking at that type of data.

CP #7, aged 73 years

Yet, when CPs actively engaged in glucose trends monitoring, they reported feeling increased peace. For example, one CP stated:

When I check, when I check my phone and for some reason there's no, no data available, I find that stressful. So then it's like, hey, how come mine's not working? So, yeah, we definitely rely on it and it gives us peace of mind.

CP #2, aged 61 years

Nonetheless, CP baseline engagement in glucose trend monitoring was mainly limited to alarm notification regarding hypoglycemic glucose trends. As part of the Share “plus” intervention, dyads were encouraged to discuss and set shared notification alerts regarding glucose levels. One person with diabetes shared:

(CP) doesn't involve herself with the highs…As I said, they're transient, the lows are more important to me. The lows are a life-threatening circumstance. The highs are not.

Person with diabetes #4, aged 66 years

While dyads set similar limits for hypoglycemic alerts, CPs set alerts for hyperglycemic trends at levels higher compared to the persons with diabetes in order to avoid or limit the alerts they received.

### Results From Postsession Interviews

#### Overview

During the postintervention interviews, similar themes emerged, which included (1) shared responsibility; (2) communication patterns; and (3) sharing and monitoring glucose data*.* Overall, while dyads addressed positive and negative communication patterns with the DCES, during postintervention interviews, participants identified that a change occurred from viewing diabetes management as an independent appraisal to feeling a sense of shared responsibility among the dyad. In addition, dyads addressed the positive influence the Share “plus” intervention had on promoting positive communication habits and monitoring glucose trends that emerged as part of the Share “plus” sessions.

#### Shared Responsibility

In contrast to the initial session, dyads reported how the Share “plus” sessions helped increase their awareness and shared responsibility around diabetes management. One CP shared:

It was almost like just confirming everything that we've done and that we established, you know, as a partner, as a, you know, husband and wife. And so, I would totally recommend (SHARE “plus”) for anybody. And I think if someone's not married and they have diabetes… that they should find that accountability partner and share that with that person… Because I think everybody should have the partnership to help them with (diabetes).

CP #1, aged 60 years

Dyads shared how an increased sense of partnership emerged as they interacted with the DCES and each other. One person with diabetes shared:

I think going through this class it kind of opened me up to the fact that because we're a partner, we're a team on this and it's just something we're facing together, it kind of made me think, “Okay” yeah.” I still primarily see it as because it's my body and my l’fe, it's primarily my issue, but it is a shared issue and I think the class kind of helped me open up to that a little bit more, so I think that's why the difference in the answer.

Person with diabetes #3, aged 67 years

CPs also described how they came to view the issue of shared responsibility through engagement with the diabetes educator session. For example, a CP stated, “Well, I just think being aware has made it really, really nice. We feel like we’re more in touch” (CP #10, aged 81 years). Through working with the DCES, dyads described becoming more open to sharing responsibility within the dyad and addressed the positive impact on the dyad relationship.

#### Communication Patterns

Upon reflecting on involvement in the Share “plus” sessions, persons with diabetes reported conversations increased around strategies to promote better support and collaboration with their CPs. One person with diabetes shared, “well we've always communicated a lot, but we communicated more about my diabetes in general because of being involved in the study, which is good” (person with diabetes #8, aged 63 years). Another person with diabetes described increased CP involvement, which resulted in increased levels of support. He stated:

I think we are sharing the burden of managing the lows better than what we were before. I think it helped us both realize this is something we both need to stay on top of.

Person with diabetes #3, aged 66 years

Overall, dyads reported that communication occurred in reaction to the current disease state of persons with diabetes as well as proactively considering how to increase the involvement and support of CPs.

CPs described the communication benefits of the Share “plus” sessions as positive and reinforcing teamwork. One stated, “I don’t know it’s through this, or just because she’s mentioned it, of being a little more patient and kinder when dealing with this” (CP #4, aged 67 years). Another shared:

I'm a scolder… Being demeaning anyway, so, yeah. I think the study was really helpful because it helped me realize that and it helped him realize that too that it is a partnership.

CP #2, aged 60 years

Yet, dyads also shared a few instances of continuing unsupportive communication. Unsupportive communication resulted from long-established communication patterns present between partners. For example, one CP stated, “The high one (alarm), the one I know was an actual high, I let my alarm on my phone nag him. He kept telling me, “Just turn it off. Turn it off.” I said, “Nope” (CP #9, aged 71 years). Instead of engaging in communication regarding the hyperglycemic events in persons with diabetes, the CP relied on the alarm to alert and influence the behavior of persons with diabetes. Moreover, some CPs reported engaging in language that they knew was to be avoided, such as blaming. One CP shared:

I guess I got upset, “Look, you’re falling, you know better than this. Why are you letting this fall?” ... I said “Why is this happening?” And I would assume that would be considered nagging, but, yeah.

CP #4, aged 67 years

As dyads experienced frustration, habitual and unsupportive communication patterns, such as blaming, emerged. In contrast to the session with the DCES, in the postinterviews, dyads were more likely to recognize and report communication strategies that were viewed as unhelpful. Dyads demonstrated increased awareness of communication patterns that were negative and that might hinder successful partnerships.

#### Sharing and Monitoring Glucose Data

Most dyads expressed an increased dyad awareness of glucose levels that occurred as a result of engaging in the sessions with the DCES. For example, one person with diabetes said:

I think (CP) feels good about being in the loop more than she was before, and it makes me feel better that she's aware, and (CP) can warn me or make sure I'm aware of where I am. So (Share “plus”) has been a positive. Because I've had diabetes for a long time.

Person with diabetes #10, aged 79 years

Similarly, a CP described a feeling of closeness as a benefit of this awareness, “Well, I just think being aware has made it really, really nice. We feel like we’re more in touch” (CP #10, aged 80 years). Another benefit was an increase in empathy, “I think I become a little more patient and understanding, that I can see where things are going” (CP #4, aged 67 years).

Importantly, dyads described that awareness increased feelings of safety. One person with diabetes shared, “Well what worked well was that it gave her (CP) peace of mind to know that she could have a window on things so to speak. And that made me feel good” (person with diabetes #8, aged 63 years). A CP described a sense of relief that hypo- and hyperglycemia was being prevented, “I was more aware because of the alarms, and his (persons with diabetes) alarms. So, I could go in and look at him, and talk to him, and see where he actually was” (CP #9, aged 71 years).

### DCES Session Feedback

During postsession interviews, dyads were asked to reflect on their experiences working with a DCES. Overwhelmingly, dyads shared positive experiences and addressed how regardless of their prior experiences and understanding about diabetes, they gained new information and felt supported. One CP shared:

(DCES) took the time to explain things. You know, if I had a question, you know’ she wasn't in a rush to kind of get through the next, whatever, learning module, or however it was structured. And you know, I think, you know, created an atmosphere where it was comfortable for persons with diabetes and I to both share, you know? And so, you know, it was a good, positive thing.

CP #5, aged 69 years

Persons with diabetes agreed, sharing:

(DCES)…knowledge base is huge, and she’s such a good teacher, and she can solve problems like nobody’s business. So, I mean, she can figure it out, and understands what’s going on, and she’s- and she listens, too.

Person with diabetes #5, aged 68 years

Overall, dyads shared how they gained new information and reported an increased sense of partnership. Moreover, dyads felt empowered and shared that communication, understanding regarding shared responsibility, and increased consideration of glucose trends were positively impacted by engaging in counseling sessions.

## Discussion

### Principal Findings

In this study, dyads identified how working with a DCES addressed a missing aspect of their current health care management. Most reported how they felt more empowered as the DCES took time to provide education, address problems and questions regarding aspects such as diet or medications, and overall supported skill development in communication. Additionally, perceptions regarding independent appraisal changed to dyads reporting feeling a shared responsibility for management.

Importantly, a key change in communication patterns emerged. Initially, dyads reported high initial confidence in their communication skills. Persons with diabetes asserted their ability to self-manage their diabetes, and CPs reported they were confident in relying on the self-management of persons with diabetes. However, during the DCES sessions, participants recognized their communication patterns and were able to identify patterns of negative behaviors and discussed during postsession interviews how they sought to change long-established communication patterns. Learning how to work together on supportive and unsupportive communication promoted a sense of teamwork for several of the dyads despite their long-term relationship and management of diabetes.

Overall, dyads shared how despite experiencing T1D for several years, they were supported by the education received during the diabetes education sessions. This finding may be related to the long duration of T1D in this sample of older adults and a lack of referrals to diabetes care and education. The standard of care for referring persons with diabetes for diabetes care and education is at diagnosis, annually, or when not meeting treatment targets, when complicating factors develop (medical, physical, and psychosocial), and when transitions in life and care occur [34]. Despite this standard of care, less than 5% of Medicare beneficiaries with diabetes and 6.8% of privately insured persons with diabetes have participated in diabetes self-management education and support services [35,36].

Prior research considering patient education programs in diabetes have established that programs that promote self-reflection, identification of risk factors, and action planning may positively influence perceptions of self-efficacy and support health behavior changes [37-39]. This was echoed in our study as the initial feedback from persons with diabetes highlighted feelings of independent appraisal, which evolved during the sessions into feelings of having a shared responsibility among the dyad. However, the influence of patient education programs encouraging persons with diabetes and their CPs to engage in CGM data sharing has not been previously considered. This is the first study to assess the experiences of dyadic participants in a telehealth patient education intervention to support CGM data sharing communication.

During the Share “plus” intervention sessions, dyads demonstrated increased appreciation for diabetes management collaboration. At first, dyads often shared beliefs that persons with diabetes retained ultimate responsibility for the management of their diabetes and that CP’s role was to provide supportive actions. However, as dyads progressed through the sessions and provided poststudy feedback, it became clear that most realized how the CP could be more involved in diabetes management, without taking over. At the end of the 12-week study, dyads reported a sense of teamwork and a shared sense of responsibility. These results are consistent with the DCM that guided the Share “plus” intervention in that changes in dyadic appraisal accompanied strategies of collaboration.

The involvement of CPs in diabetes management is especially important as persons with diabetes grow older because of the many diabetes age-related changes such as hypoglycemia unawareness and deleterious effects of hyperglycemia causing hospitalization [40]. Both hypoglycemia and hyperglycemia can also cause multiple complications including myocardial infarction, cerebral vascular accidents, seizures, and falls [41]. Especially concerning is the relationship between hypo- and hyperglycemia and dementia [42]. A proactive care model is needed for older adults with diabetes that includes support from an engaged CP or several CPs before complications such as cognitive impairment occur. Involving CP’s earlier in diabetes management may serve to provide early detection of cognitive changes and prevent life-threatening diabetes management mistakes.

Persons with diabetes who receive training in data tracking and in the use of CGM have demonstrated improved outcomes [43-45]. However, there have been few studies examining CGM with data sharing in older adults [27,46]. Moreover, family members report wanting to be more involved in care of persons with diabetes but often lack knowledge about specific diabetes management strategies or how to prevent hypo- or hyperglycemia [47,48]. Thus, family members report feeling burdened and distressed about diabetes. Recent evidence from a couples-based intervention for those with type 2 diabetes found that improving collaboration and communication resulted in lower persons with diabetes and CP distress and higher relationship satisfaction [15,49].

### Limitations

The limitations of this study include the lack of a diverse dyad sample. Moreover, further consideration of nonspousal CP relationships is necessary to explore the unique needs of other types of CP relationships. It is possible that this sample was biased as persons with diabetes and CP’s with poor relationships may not have volunteered for this study. Additionally, the findings are limited by the small sample size and the lack of racial or ethnic diversity. Further study is needed with a larger more diverse sample of dyads. Additionally, further study is needed regarding the quantitative understanding of the dyad relationship quality before and after the intervention in a larger sample.

### Conclusions

In conclusion, persons with diabetes and their CPs experienced improved communication skills and glucose management strategies after participating in the Share “plus” program. Families are often not included when addressing data-sharing with T1D despite the American Diabetes Association recommendation that CPs be involved in the care of older adults with diabetes [10]. Additionally, there has been limited training for diabetes care teams on how to provide educational or clinic visits with CPs. The Share “plus” intervention contributes to behavioral science by providing an educational curriculum to improve dyadic communication and support using CGM with data sharing. Care models are needed that actively engage persons with diabetes and CP in strategies that promote communication and problem-solving as well as CGM data sharing.

## Abbreviations

CGM: continuous glucose monitoring
CP: care partner
DCES: Diabetes Care and Education Specialist
DCM: Dyadic Coping Model
MoCA: Montreal Cognitive Assessment
T1D: type 1 diabetes
